# Isolation of Endophytic Fungi and Effects on Secondary Metabolites in Hairy Roots of *Salvia miltiorrhiza*

**DOI:** 10.4014/jmb.2411.11051

**Published:** 2025-04-09

**Authors:** Yiming Wang, Shiyu Cai, Ziling Tao, Junzhi Peng, Dan Li, Ludan Li, Xiaoying Cao, Jihong Jiang

**Affiliations:** The Key Laboratory of Biotechnology for Medicinal and Edible Plants of Jiangsu Province, School of Life Sciences, Jiangsu Normal University, Xuzhou, Jiangsu Province 221116, P.R. China

**Keywords:** Endophytes, fungi, microbial biotechnology, symbiosis, food microbiology, *Salvia miltiorrhiza*

## Abstract

The slow growth rate of medicinal plants has made them unable to meet people's needs, and the use of biotechnology to obtain natural products from medicinal plants can alleviate this problem. This study isolated and identified 42 endophytic fungi from the roots, stems, and leaves of *Salvia miltiorrhiza*, belonging to 13 genera. The endophytic fungi that promote the accumulation of secondary metabolites in the hairy roots of *S. miltiorrhiza* were screened by co-culture and elicitors preparation. Among them, 15 endophytic fungi presented relatively high crude polysaccharide yields. Co-culture experiments showed that endophytic strains had different effects on the biomass and the accumulation of secondary metabolites in the hairy roots of *S. miltiorrhiza*, with strain KLBMPSM237 being the most effective. The contents of tanshinone I, salvianolic acid B and rosmarinic acid in the hairy roots of *S. miltiorrhiza* were significantly increased by KLBMPSM237 polysaccharide inducers at different concentrations. This study provides new microbial resources and technical methods for increasing the natural products in hairy roots of *S. miltiorrhiza*.

## Introduction

*Salvia miltiorrhiza*, a medicinal plant of the Salvia genus in the Labiatae family, has the effects of promoting blood circulation, removing blood stasis, regulating menstruation, relieving pain, clearing the heart and cooling the blood, and eliminating irritation and carbuncles [1-4]. It has a long history as a traditional Chinese medicine in China [5, 6]. Its main components include water-soluble components (such as salvianic acid A) and fat-soluble components (such as tanshinone I), which are commonly used to treat cardiovascular and cerebrovascular diseases, chronic liver disease, cancer, and osteoporosis [7-10]. At present, the main source of *S. miltiorrhiza* medicinal materials is artificial cultivation, which is difficult to control the quality and varies greatly in quality from region to region [11, 12]. With the increase in the incidence of cardiovascular diseases in recent years, the demand for *S. miltiorrhiza* medicinal materials has also exceeded the supply.

Endophytic fungi are widely present in plants, and refer to fungi that exist in the living tissues of plants during some or all stages of their life cycle, and do not cause significant disease in the plant itself [13-15]. They also achieve a dynamic balance of growth with their host to some extent, forming a mutually beneficial relationship [16-18]. Some endophytic fungi can also positively regulate the biosynthesis of plant secondary metabolites through polysaccharide elicitors [19, 20]. Hairy roots are a high-quality source of active ingredients in medicinal plants, with the advantages of rapid growth, yields of secondary metabolites similar to those of plant, and high genetic stability [21-23]. The use of hairy root technology to improve the quality of medicinal plants and obtain secondary metabolites from medicinal plants has gradually become a research hotspot.

This study isolated endophytic fungi from *S. miltiorrhiza* and cultured them with hairy roots of *S. miltiorrhiza* to find functional dominant strains that promote the content of secondary metabolites in *S. miltiorrhiza*, providing microbial technology support for improving the production of natural products from *S. miltiorrhiza*.

## Materials and Methods

### Isolation and Purification of Endophytic Fungi from *S. miltiorrhiza*

The roots, stems, and leaves of *S. miltiorrhiza* were taken and rinsed with running water. The surface was disinfected with ethanol and 8% sodium hypochlorite and then rinsed twice with sterile water. *S. miltiorrhiza* was cut into small pieces of 0.5 × 0.5 cm and placed in a constant temperature incubator at 28°C with sterile tweezers on the separation medium (Tsaberg's medium, Martin's AGAR medium, Wickerham's medium and Sharpie's medium), and the last rinse of water was coated in the medium as a control and incubated for 7 days. The endophytic fungi were transferred from tissues to PDA medium for culture.

### Identification of Endophytic Fungi in *S. miltiorrhiza*

The DNA of endophytic fungi in *S. miltiorrhiza* was extracted by CTAB method [24]. The DNA was subsequently used as a template for PCR amplification with universal primers ITS4 (5'-TCCTCCGCTTATTGATATATGC-3') and ITS5 (5'-GGAAGTAAAAGTCGTAACAAGG-3'). The enzyme used in the PCR reaction was NaviScript^®^ Rapid PCR Master Mix (Synomebio Co., Ltd., China) and the reaction system and reaction conditions refer to the product description. After electrophoresis of the PCR products, the target fragment was recovered by gel cutting, cloned and sequenced by Sangon Bioengineering (China). The sequencing results were compared with the relevant strain sequences in NCBI (https://www.ncbi.nlm.nih.gov/) to determine the types of endophytic fungal strains. The gene sequences of the strains have been uploaded to NCBI GenBank (Accession No. PQ533214-PQ533226).

### Extraction and Content Determination of Extracellular Crude Polysaccharides from the Endophytic Fungi of *S. miltiorrhiza*

Extraction of polysaccharides from endophytic fungi was optimized based on Naveen *et al*.'s method [25]. The liquid fermentation products of each strain were centrifuged at 5,000 r/min for 15 min, and the mycelia were discarded. The supernatant was mixed with three times its volume of 95% ethanol, allowed to settle overnight, filtered, and discarded. Then, three times volumes precipitation water was added for redissolution, followed by centrifugation at 5,000 r/min to remove insoluble impurities. The Sevag method (chloroform:n-butanol = 4:1) was employed to eliminate soluble protein impurities from the sample mixture [25, 26]. After vigorous stirring for 20 min, the mixture was subjected to static stratification in a separation funnel and the lower layer was discarded. This process was repeated multiple times to eliminate free proteins from the polysaccharide solution. Following protein removal, the polysaccharide sample solution was concentrated by a rotary evaporator under reduced pressure; n-butanol miscible with water vaporized during this process. An appropriate amount of water was then added for redissolution 3.5 times before its volume of 95% ethanol was added overnight sedimentation at 4°C. The resulting precipitate was filtered and washed twice with ethanol until all residual ethanol had evaporated to obtain the crude polysaccharide. The polysaccharide content of the product was determined by phenol-sulfuric acid method [26].

### Effects of High Polysaccharide-Producing Endophytic Fungi on Hairy Root Growth and Secondary Metabolites of *S. miltiorrhiza*

Fungal and hairy root co-cultures were performed using the method of Xie *et al*. [22]. The 0.3 g of hairy roots of *S. miltiorrhiza* were inoculated into 250 ml flasks containing 100 ml of 1/2 MS medium and incubated in a constant temperature shake flask (25°C, 120 rpm) in the dark. Meanwhile, endophytic fungi producing *S. miltiorrhiza* polysaccharides were inoculated on PDA solid medium and cultured at 28°C. When the medium was changed for the third time, the hairy roots were randomly divided into an experimental group and a control group. The endophytic fungi were then punched into fungal cakes with a diameter of 5 mm and inoculated into the hairy root culture medium of the experimental group, while the control group received an equal amount of PDA solid medium. Each experiment was repeated three times. After five days, growth was recorded, followed by separation and weighing of the hairy roots which were subsequently dried at 50°C.

Six standard substances (rosmarinic acid, salvianolic acid B, tanshinone I, Tanshinone IIA, dihydrotanshinone I, cryptotanshinone) were prepared into 1 mg/ml stock solution and further diluted 10 to 100 times. The dried hairy roots of *S. miltiorrhiza* were powdered and accurately weighed (0.10 g) in a 15 ml centrifuge tube. Then, methanol (2 ml) was added to the tube via a pipette gun and allowed to soak for 2 h. Simultaneously, an ultrasonic instrument was heated to 60°C for extraction through ultrasonication for one hour. After filtration with a sterilized filter head (0.22 μm), the sample solution was obtained and prepared accordingly. Samples were sent to Nanjing Jiangbei New Area Sci-tech Investment Group for quantitative detection. For high-performance liquid chromatography analysis using an Agilent-1200 system equipped with a Halo C18-OL chromatographic column (2.1 × 150 mm; particle size: 2.7 μm), the mobile phase consisted of A: aqueous solution containing formic acid (0.2%) and B: acetonitrile with gradient elution conditions set as follows: within sixty minutes increase from initial B ratio of ten percent to one hundred percent maintained for twenty minutes; flow rate set at 0.4 ml/min; detection the wavelength of DAD detector were set at 254 nm, 270 nm, and 320 nm respectively. Finally, the relative content was calculated using the external standard method. The functional dominant strains were screened based on changes in the main product content.

### Effects of the KLBMPSM237 Elicitor on the Growth and Secondary Metabolism of *S. miltiorrhiza* Hairy Roots

Endophytic fungus KLBMPSM237 was inoculated into potato dextrose liquid medium and incubated for 7 days at 28°C with agitation at 130 rpm. After vacuum filtration, mycelia were collected and washed three times with distilled water. Subsequently, each gram of mycelia was homogenized in 15 ml of distilled water for five minutes, subjected to 1 h ultrasonic treatment, and finally treated at 121°C for 30 min. After another round of vacuum filtration, the resulting filtrate was considered the KLBMPSM237 elicitor solution.

The sugar concentration in the induction solution was determined by measuring glucose levels using an anthrone-sulfate colorimetric assay and constructing a standard curve [27]. The absorbance value was plotted with the corresponding standard glucose content (μg) as the horizontal axis to construct the standard curve.

The KLBMPSM237 elicitor solution (2 ml) was drawn and carefully added to another 50 ml volumetric flask containing distilled water. After thorough shaking, 1 ml of the mixture was transferred into an empty test tube, followed by the addition of anthrone reagent (4.0 ml). Use an equal volume of water as a contrast. Subsequently, the absorbance at a wavelength of 620 nm was measured, and the concentration of KLBMPSM237 inducible sugar was calculated using the glucose standard curve equation.

The hairy roots of *S. miltiorrhiza* (0.5 g) were inoculated into 250 ml conical flasks containing 100 ml of 1/2 MS culture medium and cultured at 25°C and 120 rpm in the dark. The medium was refreshed every 6 days. During the third medium change, hairy roots were randomly divided into three groups according to the experimental method of Xu *et al*. [28]: control group, low concentration elicitor group (50 mg/l), and high concentration elicitor group (150 mg/l). The same volume of 1/2 MS medium was added to the control group's conical flask with three replicates for each group. Samples were collected at day 6, day 12, and day 18, respectively. The others were kept on the medium containing the inducer and changed every 6 days. The samples were filtered under reduced pressure, weighed for fresh weight measurement, and dried in an oven at a constant temperature of 50°C until reaching a constant weight. Sample solutions were prepared using the same method and analyzed by HPLC.

### Data Analysis

Graphpad software and Microsoft Excel were used for data analysis and picture creation, and Microsoft Word was used to create tables. Images were processed using Adobe Illustrator. Single-factor analysis of variance (ANOVA) was used to evaluate the differences in the data obtained from the experiment, with a significance level set at *p* < 0.05. All experiments were repeated at least three times.

## Results and Discussion

### Isolation and Identification of Endophytic fungi from *S. miltiorrhiza*

**Fig. 1:**
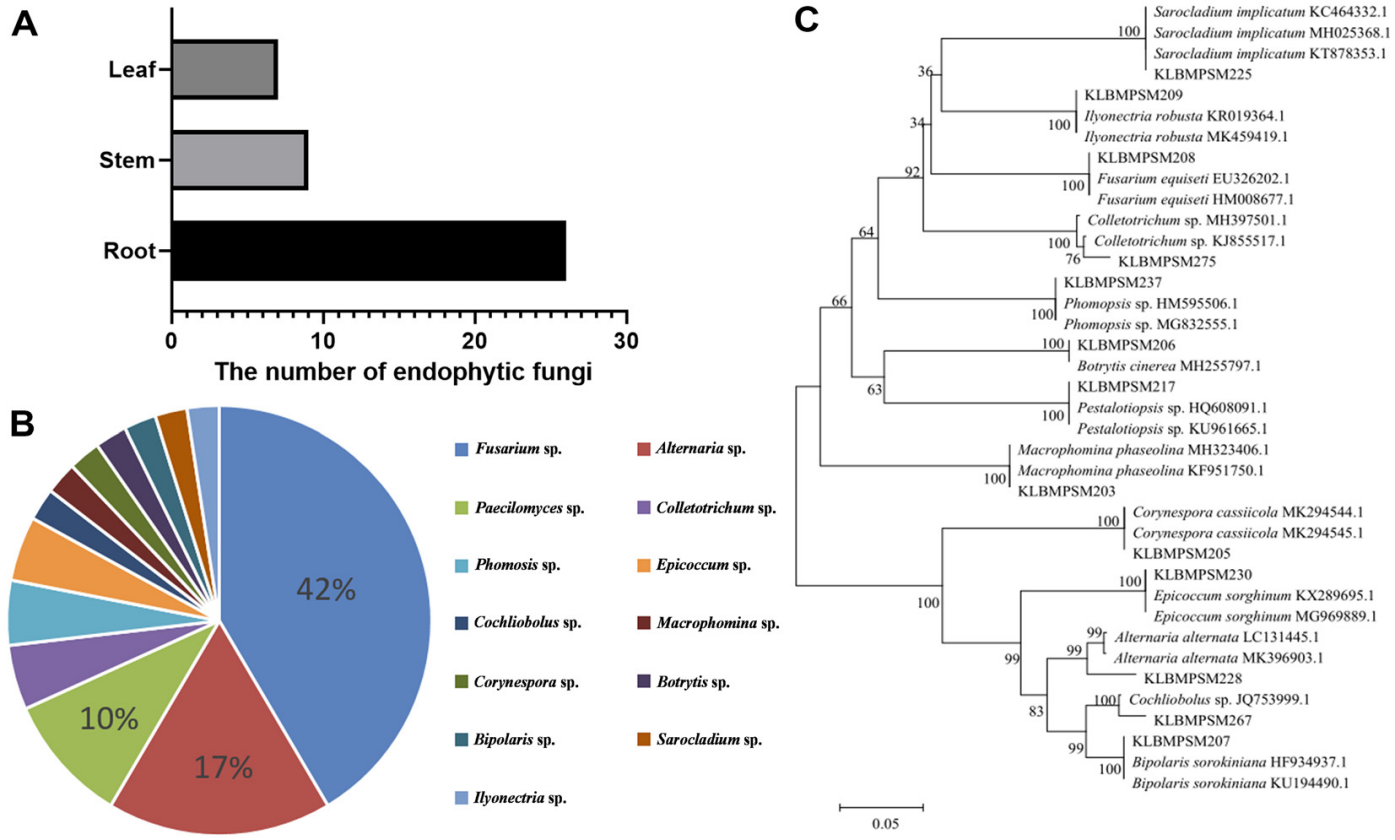
(A) Number of endophytic fungi isolated from different parts of *S. miltiorrhiza*. (B) Diversity of endophytic fungi. (C) Phylogenetic tree of endophytic fungi.

After 7 days of culture at 28°C, no microorganisms were observed on the test medium, indicating a superior surface sterilization effect of *S. miltiorrhiza* plant tissue. A total of 42 endophytic fungul strains were isolated from the roots, stems, and leaves of *S. miltiorrhiza*. Statistical analysis revealed that the highest number of endophytic fungi strains was isolated from roots (26 strains), accounting for 62% of the total number. This was followed by stems, while leaves had the lowest number with only 7 strains, representing 17% of the total (Fig. 1A).

42 strains of endophytic fungi from *S. miltiorrhiza* were subjected to DNA extraction and PCR amplification of ITS sequences, and preliminary classification was performed based on molecular identification, a total of 13 genera. Among them, Fusarium was the dominant strain in the isolation experiments, accounting for 42% of the total strains (Fig. 1B). Phylogenetic trees were constructed by maximum likelihood using MEGA 11, and 1000 bootstrap replicates were performed to assess tree robustness (Fig. 1C).

### Determination of Polysaccharide-Producing Ability of Endophytic Fungi

On the basis of the similarity of the ITS sequences among the strains, repetitive strains were excluded from a total of 42 endophytic fungi, resulting in the selection of 30 endophytic fungi from *S. miltiorrhiza* for evaluating their polysaccharide production capacity. The findings indicated variability in crude polysaccharide yields across different strains. Notably, KLBMPSM246, KLBMPSM220, KLBMPSM254, KLBMPSM275, KLBMPSM273, KLBMPSM217, KLBMPSM248, KLBMPSM261, SM236, KLBMPSM267, KLBMPSM215, and others produced approximately 0.3 g/l of fermentation broth. Among these strains, *Alternaria* sp. KLBMPMS234 exhibited the highest sugar yield followed by *Fusarium* sp. KLBMPMS229 and *Phomopsis* sp. KLBMPMS237 (Table 1).

### Effects of Co-Culture on Hairy Root

**Fig. 2:**
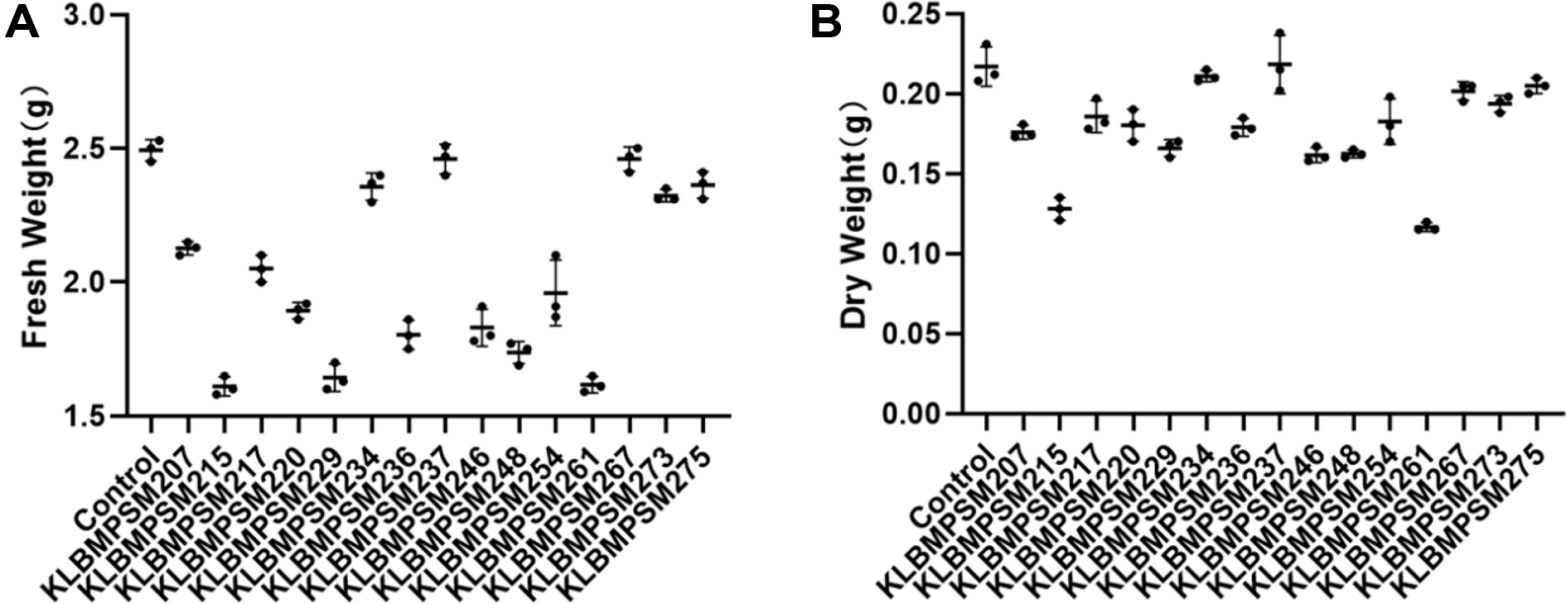
Effects of the endophytic fungi on the hairy root biomass of S.miltiorrhiza. (**A**) Dry weight. (**B**) Fresh weight (The number of replicates: *n* = 3).

After co-culturing with hairy roots of *S. miltiorrhiza* for 5 days using fifteen selected endophytic fungal strains significant differences were observed in the effects of different endophytic fungi on the biomass of hairy roots (Fig. 2): compared with the control group, strains such as KLBMPSM215, KLBMPSM229, and KLBMPSM261 significantly reduced the biomass of hairy roots; The fresh and dry weight of hairy roots inoculated with KLMBPMS237 strain were comparable to the control group.

### Effects of Co-Culture on the Secondary Metabolism of Hairy Roots

**Fig. 3:**
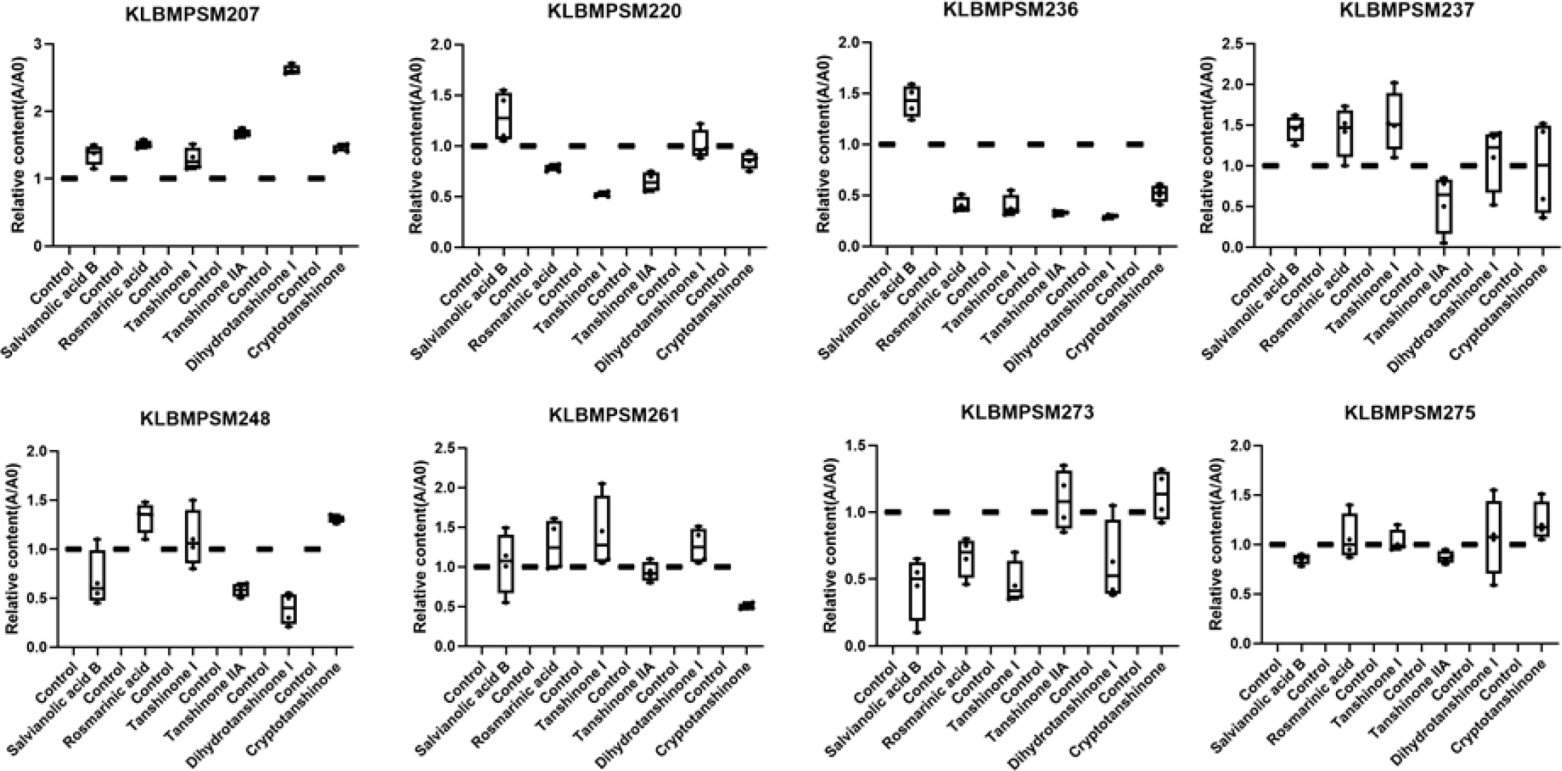
Effects of endophytic fungi on the components of hairy roots of *S. miltiorrhiza* (A, absorbance of substance in experimental group; A0, absorbance of substance in control group; The number of replicates: *n* = 4).

The six metabolites: rosmarinic acid, salvianolic acid B, tanshinone I, tanshinone IIA, dihydrotanshinone I, and cryptotanshinone identified in hairy roots inoculated with strain KLBMPSM246 were found to be significantly lower than those present in the control; Similarly, five metabolites associated with hairy roots inoculated with both KLBMPSM236 and KLBMPSM220 were lower than those associated with the control; In addition, four metabolites in the hairy roots of *S. miltiorrhiza* inoculated with strains KLBMPSM273 and KLBMPSM217 were lower than those in the control; Furthermore, three metabolites in the hairy roots of *S. miltiorrhiza* inoculated with strains KLBMPSM248, KLBMPSM234, and KLBMPSM215 also showed decreased levels relative to controls. Notably, more than four metabolites from *S. miltiorrhiza* hairy roots inoculated with strains KLBMPSM237, KLBMPSM261, KLBMPSM267, KLBMPSM275, KLBMPSM207 and KLBMPSM254 were elevated compared to the control; Among these strains, KLBMPSM207 and KLBMPSM237 demonstrated particularly pronounced effects (Fig. 3). From a biomass perspective, the fresh weight and dry weight of strain KLBMPSM207 were reduced by 18% and 26%, respectively, in comparison to the control after co-cultivation with the hairy roots of *S. miltiorrhiza*. Conversely, strain KLBMPSM237 did not exert a significant impact on biomass following co-culture with the hairy roots of *S. miltiorrhiza*. Therefore, the KLBMPSM237 strain was targeted for further studies.

### Effects of the KLBMPSM237 Inducer in Hairy Root Growth and Secondary Metabolism

The standard curve was drawn with the concentration of glucose solution as the abscissa and the absorbance as the ordinate. The standard curve equation was Y = 0.0081X - 0.0111, R^2^ = 0.9942. The OD_620_ of the elicitor solution was 0.125, and the sugar concentration of the elicitor solution was calculated to be 0.42 mg/ml according to the standard curve equation.

**Fig. 4:**
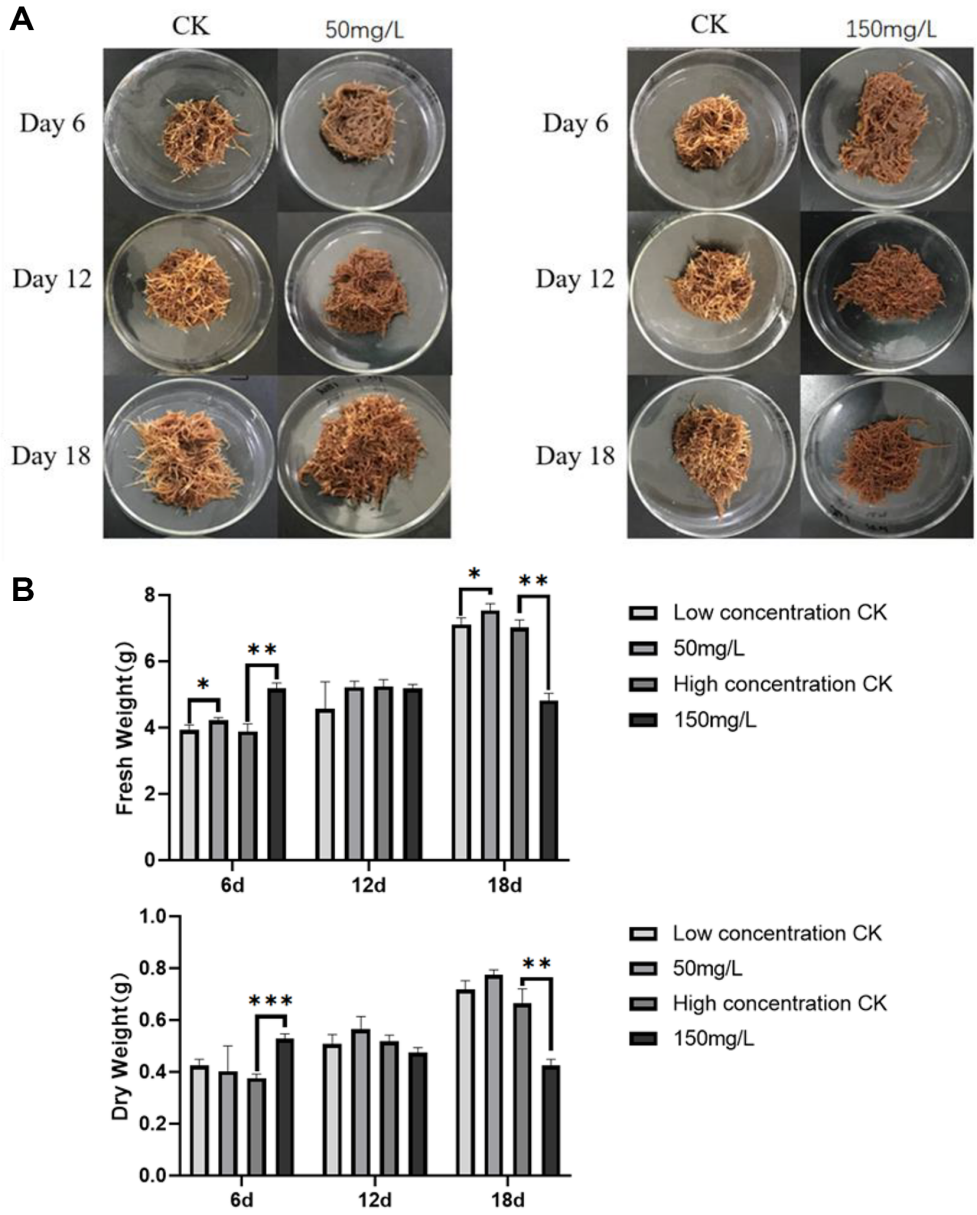
(A) Growth of *S. miltiorrhiza* hairy roots at different times and different KLBMPSM237 elicitor concentrations (B) Changes in the biomass of *Salvia miltiorrhiza* hairy roots at different time and different KLBMPSM237 elicitor concentrations (* represents *p* < 0.05, * * represents *p* < 0.01, * * * represents *p* < 0.001 vs the corresponding control group; The number of replicates: *n* = 3).

The mycelia of *S. miltiorrhza* from the endophytic fungus KLBMPSM237 were harvested post-fermentation, and two polysaccharide concentration elicitor solutions (50 mg/l and 150 mg/l) were formulated and introduced into the cultured hairy root medium of *S. miltiorrhza* under the influence of the KLBMPSM237 elicitor. Both concentrations made the hairy tissue and medium of *S. miltiorrhiza* appear red and darker in the high concentration group (Fig. 4A).

The biomass of *S. miltiorrhiza* hairy roots under the role of the KLBMPSM237 elicitor is shown in Fig. 4B. On day 6, a high concentration of the KLBMPSM237 elicitor significantly enhanced the fresh weight and dry weight of *S. miltiorrhiza* hairy tissues while a low concentration resulted in a significant increase in fresh weight. On day 18, the low concentration also led to a significant increase in fresh weight for *S. miltiorrhiza*, whereas the high concentration significantly inhibited both fresh weight and dry weight.

**Fig. 5:**
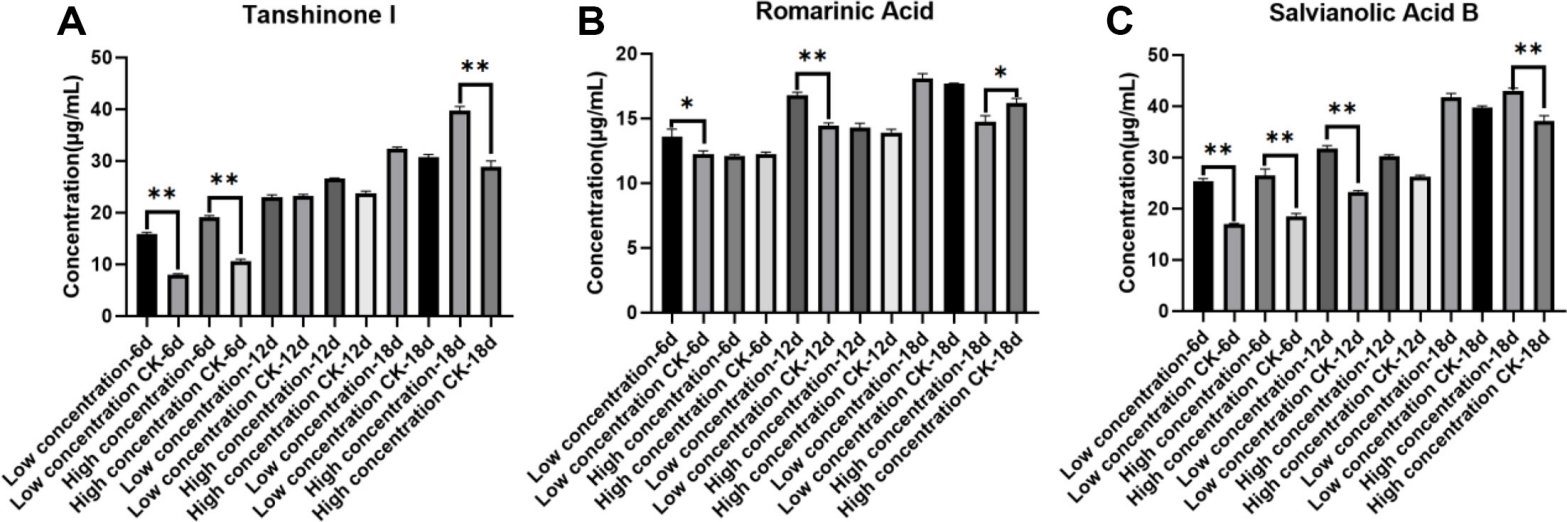
Effect of different KLBMPSM237 elicitor concentrations on the active constituents of *S. miltiorrhiza* hairy roots. (**A**) Tanshinone I; (**B**) Rosmarinic acid; (**C**) Salvianolic acid B (* represents *p* < 0.05, ** represents *p* < 0.01; The number of replicates: *n* = 3).

The influence of the KLBMPSM237 elicitor on the secondary metabolism of hairy roots from *S. miltiorrhiza* is depicted in Fig. 5. Following the application of KLBMPSM237, significant increases in tanshinone I content were observed by day 6, with low and high concentrations resulting in enhancements of 82% and 71%, respectively, compared with those of the controls. Although the changes in content became less pronounced by day 12, the levels remained elevated relative to the control. By day 18, both low and high concentrations of KLBMPSM237 continued to significantly elevate tanshinone I levels in the hairy roots, yielding a concentration that was 26%higher than that observed in the control (Fig. 5A).

On day 6, there was no evident trend regarding the effect of KLBMPSM237 on rosmarinic acid content; however, by day 12, low concentrations of KLBMPSM237 increased rosmarinic acid levels in hairy roots of *S. miltiorrhiza* by 19% compared to controls, while no significant difference was noted between the high concentration experimental group and the control group. On day 6 and 18, high concentrations of KLBMPSM237 inhibited the rosmarinic acid content (Fig. 5B).

The content of salvianolic acid B significantly increased due to both high and low concentrations of KLBMPSM237 at each time point measured. On day 6, salvianolic acid B levels were found to be elevated by 53%and 45% at low and high concentrations respectively compared with controls. By day 12, these elicitors further increased salvianolic acid B contents by approximately 24% for low concentration and about 16% for high concentration groups relative to controls. On day 18, both low and high concentrations of KLBMPSM237 significantly enhanced the content of salvianolic acid B in the hairy roots of *S. miltiorrhiza*, with increases of 17%and 23%, respectively, incomparison to the control (Fig. 5C).

## Discussion

*S. miltiorrhiza* related preparations are in high demand worldwide because of their significant therapeutic effects on cardiovascular and cerebrovascular diseases [29-31]. However, the current production of *S. miltiorrhiza* is mostly cultivated, with a growth cycle of up to 2 years, and the quality varies in different production areas [32-35]. Therefore, it is necessary to use modern scientific and technological methods to improve traditional cultivation techniques to obtain high-quality *S. miltiorrhiza* and medicinal natural products. Because hairy roots have the advantages of rapid growth, high yields of secondary metabolites, and high genetic stability, hairy roots of medicinal plants have been applied to the production of secondary metabolites and for variety improvement.

Endophytic fungi are commonly found in healthy plant tissues [36, 37]. Previous studies have shown that endophytic fungi can promote the growth and development of medicinal plants and the accumulation of secondary metabolites [16, 19]. Wu *et al*. co-cultured the mangrove plant *Rhizophora mangle* with Phomopsis fungus, resulting in the production of a series of alkaloids, sterols, and polyketones [10]. *Beauveria bassiana*, the endophytic fungus of tomato, can also induce the production of alkaloids, flavonoids and other substances in tomato, thus playing a role in insect resistance[38]. Endophytic fungi can be made into biological inducers to activate the expression of genes in secondary metabolic pathways and promote the accumulation of active ingredients in medicinal plants [19, 39]. Liu *et al*. screened three strains of fungi with a growth promoting function from *Taxus chinensis* and used fermentation broth to prepare elicitors to significantly increase Taxane accumulation in *T. Chinensis* stem cells [40]. Wang *et al*., isolated the endophytic fungus *Aspergillus Niger* from the endothelium of yew trees and prepared an inducer, stimulated Taxol production in suspension cultures of yew cells [41].

In this study, a total of 42 endophytic fungi were isolated from *S. miltiorrhiza*. A strain *Phomopsis* sp. KLBMPSM237 that promoted the accumulation of active components in hairy roots of *S. miltiorrhiza* without biomass inhibition was selected from the crude polysaccharide production of endogenous fungi and the co-cultivation of hairy roots. Different concentrations of inducers produced by the strain significantly increased tanshinone I salvianolic acid B and biomass of hairy roots of *S. miltiorrhiza*. Tanshinone I and salvianolic acid B are the important active components in *S. miltiorrhiza* [42, 43]. Tanshinone I has good activities, including anti-oxidative stress, regulating autophagy or apoptosis, and inhibiting inflammation [44]. Salvianolic acid B is a water-soluble weakly acidic drug that has been shown to have anti-tumor and anti-inflammatory effects on various organs and tissues such as lung, heart, kidney, intestine, bone, liver, and skin, as well as protective effects on diseases such as depression and spinal cord injury [45]. It is important for the pharmaceutical industry to be able to use biotechnology to improve the production of pharmaceutical ingredients. The precursor substances involved in the metabolism of *S. miltiorrhiza* are catalyzed by the enzyme genes of CYP98A family to produce rosmarinic acid, which is then used as the precursor of salvianolic acid B [46]. This implies that the reduction of rosmarinic acid may be involved in the production of salvianolic acid B. The metabolism of tanshinone IIA, cryptotanshinone (the precursor of tanshinone IIA) and tanshinone I are in different branches under the catalysis of CYP76A and CYP81A family genes [47]. The increase of tanshinone I and the decrease of tanshinone IIA suggest that there may be a competitive relationship among them. This may be the potential mechanism by which KLBMPSM237 promotes the accumulation of tanshinone I and salvianolic acid B in the hairy roots of *S. miltiorrhiza* and improves its medicinal value.

At present, the effects of endophytic fungi on host medicinal plants are not well studied. Some studies have shown that endophytic fungi regulate the secondary metabolism of medicinal plants by regulating signalling molecules, enzyme genes, and plant hormones in plants, thereby improving the quality of medicinal plants[19, 48-50]. This study provides new microbial resources for improving the composition of tanshinone I and salvianolic acid B in the hairy roots of *S. miltiorrhiza*. However, the exact pathway by which *Phomopsis* sp. KLBMPSM237 promotes the growth and accumulation of active components in *S. miltiorrhiza* remains to be further studied.

## Conclusion

In this study, a *Phomopsis* sp. KLBMPSM237 strain was selected from the medicinal plant *S. miltiorrhiza*, which promoted the accumulation of active components in the hairy roots of *S. miltiorrhiza* without biomass inhibition. The results showed that different concentrations of the inducer significantly increased tanshinone I, salvianolic acid B and the biomass of hairy roots of *S. miltiorrhiza*. This study provides a new biotechnology support for promoting the production of essential ingredients from hairy root of *S. miltiorrhiza*.

## Supplemental Materials

Supplementary data for this paper are available on-line only at http://jmb.or.kr.



## Figures and Tables

**Table 1:** The ability of endophytic fungi to produce polysaccharides from *S. miltiorrhiza* (The number of replicates: *n* = 3, take the average value).

Number	Species name	Polysaccharide (g/L)	Number	Species name	Polysaccharide (g/L)
KLBMPSM201	*Fusarium* sp.	0.055	KLBMPSM228	*Alternaria* sp.	0.183
KLBMPSM202	*Epicoccum* sp.	0.107	KLBMPSM229	*Fusarium* sp.	0.596
KLBMPSM205	*Corynespora* sp.	0.167	KLBMPSM231	*Alternaria* sp.	0.195
KLBMPSM206	*Botrytis* sp.	0.251	KLBMPSM233	*Alternaria* sp.	0.200
KLBMPSM207	*Bipolaris* sp.	0.486	KLBMPSM234	*Alternaria* sp.	1.324
KLBMPSM208	*Fusarium* sp.	0.190	KLBMPSM235	*Fusarium* sp.	0.195
KLBMPSM210	*Alternaria* sp.	0.210	KLBMPSM236	*Phomopsis* sp.	0.377
KLBMPSM213	*Fusarium* sp.	0.063	KLBMPSM237	*Phomopsis* sp.	0.587
KLBMPSM214	*Fusarium* sp.	0.119	KLBMPSM246	*Fusarium* sp.	0.304
KLBMPSM215	*Fusarium* sp.	0.461	KLBMPSM248	*Fusarium* sp.	0.362
KLBMPSM217	*Pestalotiopsis* sp.	0.321	KLBMPSM254	*Fusarium* sp.	0.305
KLBMPSM220	*Fusarium* sp.	0.305	KLBMPSM261	*Fusarium* sp.	0.366
KLBMPSM223	*Pestalotiopsis* sp.	0.047	KLBMPSM267	*Cochliobolus* sp.	0.385
KLBMPSM225	*Sarocladium* sp.	0.150	KLBMPSM273	*Fusarium* sp.	0.320
KLBMPSM227	*Alternaria* sp.	0.282	KLBMPSM275	*Cochliobolus* sp.	0.317
